# Increased Cell-Matrix Adhesion upon Constitutive Activation of Rho Proteins by Cytotoxic Necrotizing Factors from *E. Coli* and *Y. Pseudotuberculosis*

**DOI:** 10.1155/2012/570183

**Published:** 2012-07-05

**Authors:** Martin May, Tanja Kolbe, Tianbang Wang, Gudula Schmidt, Harald Genth

**Affiliations:** ^1^Institute for Toxicology, Hannover Medical School, Carl-Neuberg-Str. 1, 30625 Hannover, Germany; ^2^Institute for Experimental and Clinical Pharmacology and Toxicology, University of Freiburg, 79104 Freiburg, Germany

## Abstract

Cytotoxic necrotizing factors (CNFs) encompass a class of autotransporter toxins produced by uropathogenic *E. coli* (CNF1) or *Y. pseudotuberculosis* (CNFy). CNF toxins deamidate and thereby constitutively activate RhoA, Rac1, and Cdc42. In this study, the effects of CNF1 on cell-matrix adhesion are analysed using functional cell-adhesion assays. CNF1 strongly increased cell-matrix binding of suspended Hela cells and decreased the susceptibly of cells to trypsin-induced cell detachment. Increased cell-matrix binding was also observed upon treatment of Hela cells with isomeric CNFy, that specifically deamidates RhoA. Increased cell-matrix binding thus appears to depend on RhoA deamidation. In contrast, increased cell spreading was specifically observed upon CNF1 treatment, suggesting that it rather depended on Rac1/Cdc42 deamidation. Increased cell-matrix adhesion is further presented to result in reduced cell migration of adherent cells. In contrast, migration of suspended cells was not affected upon treatment with CNF1 or CNFy. CNF1 and CNFy thus reduced cell migration specifically under the condition of pre-established cell-matrix adhesion.

## 1. Introduction

Cell-matrix adhesion involves several processes including integrin binding, cell spreading, and flattening against the substrate. Cultured cells, that spread out on ligand coated surfaces, rearrange their cytoskeleton and begin to move. Integrins thereby cluster together in “focal complexes” at the leading edge. These focal complexes grow into mature focal contacts, also called focal adhesions (FAs) [1]. Focal adhesions contain over 100 different proteins, including integrins, adapter proteins, and intracellular signaling proteins. Clustered integrins anchor actin filaments to the cell membrane and link them with the extracellular matrix (ECM) through adapter proteins such as talin and vinculin. The adapter protein paxillin links integrins to signaling proteins, forming a scaffold for Src kinases, the focal adhesion kinase (FAK), or the p21-activated kinase (PAK) [2–5].

The turnover of FAs in moving cells is driven by small GTPases of the Rho subfamily. FA formation and disassembly at the leading edge is driven by Rac1 and the localized suppression of Rho activity. Disassembly of FAs at the cell rear requires RhoA activity [6]. The activity of Rho proteins is regulated by the GTPase cycle. Rho proteins are active in the GTP-bound state and inactive in the GDP-bound state. In their active conformation Rho proteins interact with effector proteins to transmit downstream signaling. The cycling between these states is governed by guanine nucleotide exchange factors (GEF) and GTPase activating proteins (GAP), which catalyse the exchange of GDP to GTP or stimulate the intrinsic GTP hydrolase, respectively. A critical amino acid for GAP-induced as well as for intrinsic GTPase activity is Gln-63 in RhoA (Gln-61 in Rac1 and Cdc42). Gln-63/61 is deamidated by cytotoxic necrotizing factors (CNF), a class of autotransporter toxins produced by uropathogenic *E. coli* (CNF1-3) or *Y. pseudotuberculosis *(CNFy) [7, 8]. Deamidation results in inhibition of GAP-induced as well as of intrinsic GTPase activity, resulting in so called “constitutively active” Rho proteins. CNF1-induced activation of Rho proteins leads to the assembly of F-actin in prominent stress fibers, membrane ruffles, and filopodia [9]. CNF1 triggers cell spreading of monocytes [10] and epithelial cells [11]. In CNF1-treated epithelial cells, cell spreading was accompanied by the formation of vinculin-positive FAs and the phosphorylation of FA proteins, including the focal adhesion kinase (FAK) and paxillin [12]. CNF1 exhibited antiapoptotic activity, which has been attributed to its capability of inducing cell spreading [13]. CNF1-treated cells finally acquire a multinucleated phenotype, which seems to be based on inhibited cytokinesis with ongoing cell cycle progression [14].

In this study, the effects of CNF1 on cell-matrix adhesion are analysed using functional cell adhesion assays. CNF1 strongly increases cell-matrix adhesion of suspended Hela cells and decreased the susceptibly of cells to trypsin-induced cell detachment. Increased cell-matrix adhesion is further presented to contribute to reduced cell migration. Increased cell-matrix binding is also observed upon treatment of Hela cells with isomeric CNFy, that specifically deamidates RhoA [15]. Increased cell-matrix binding appears to depend on RhoA deamidation.

## 2. Materials and Methods

### 2.1. Materials

The following reagents were obtained from commercial sources: DAPI (40.6-diamidino-2-phenylindole) (Serva), Hoechst 33342 (Cambrex), rhodamine-conjugated phalloidin (Sigma). The following reagents were obtained from commercial sources: RhoA (mAb-26C4), Rac1 (mAb-23A8) (Santa Cruz); *β*-actin (mAb AC-40) (Sigma); Cdc42 (mAb-44) (BD Transduction Laboratories); pS144/141-PAK1/2 (mAb EP656Y) (Abcam); vinculin (mAb hVIN-1) (Abcam); horseradish peroxidise-conjugated secondary antibodies (Rockland); anti-rabbit IgG Alexa Fluor 488 goat secondary antibody (Invitrogen).

CNF1 from *E. coli* and CNFy from *Y. pseudotuberculosis* were expressed as GST fusion proteins in *E. coli* and purified by affinity chromatography using glutathione-sepharose, as described earlier [15]. Toxin B (TcdB) from *Clostridium difficile* strain VPI10463 was purified as described [16].

### 2.2. Cell Culture and Transwell Migration Assay

HeLa cells were maintained in Dulbecco's minimal essential medium supplemented with 100 *μ*g/mL penicillin, 100 U/mL streptomycin, 1 mM sodium pyruvate, and 10% FCS at 37°C in a humidified atmosphere containing 5% CO_2_. The migration assay was performed in a Boyden chamber using 8 *μ*m pore diameter 24-well transwell filter inserts (Corning). Suspended cells were directly placed on the membrane or allowed to adhere for 2 h. Cell migration was stimulated by FCS, present in the bottom solution for 5 h. Cells on the upper membrane surface were scraped with a cotton swab, and cells on the bottom surface stained with Hoechst 33342. After incubation at 37°C for 15 min, cells were analyzed by fluorescence microscopy using a Zeiss Axiovert 200 M (excitation 365 nm, emission 420 nm). Five adjacent microscope fields for each membrane were counted at ×20 magnification.

### 2.3. Cell-Matrix Binding Assays

Establishment of cell-matrix binding of suspended cells: HeLa cells were treated with the indicated toxin for 2 h and suspended by trypsin. Suspended cells were seeded onto cell culture dishes (polystyrol) coated with or without fibronectin. At the indicated time points, the medium containing nonadherent cells was removed, and attached cells were documented by phase contrast microscopy. Susceptibility towards trypsin-induced cell detachment: adherent HeLa cells were treated with the indicated toxin for 2 h. Cells were incubated with trypsin for the indicated time and the medium with suspended cells was removed. Adherent cells were documented by phase contrast microscopy. Five adjacent microscope fields for each membrane were counted at ×20 magnification.

### 2.4. Immunocytochemistry and Immunofluorescence

4 × 10^5^ HeLa cells were seeded onto cover slides and treated as indicated. Cells were fixed in 3.7% paraformaldehyde for 10 min at room temperature. The samples were washed with PBS and incubated with 5% BSA in PBS at room temperature for 1 h. Primary antibody was diluted in PBS and samples were incubated at room temperature for 1 h. Subsequently, cells were incubated with an Alexa Fluor 488 conjugated secondary antibody diluted to 1 : 1000 in PBS and DAPI (1 *μ*g/mL) for 1 h at room temperature. Cover slides were fixed using prolong Antifade (Invitrogen) and analysed by fluorescence microscopy using Leica confocal microscope Inverted-2.

### 2.5. Rho Effector Pull-Down Assay

Effector pull-down assay were performed using the Rho binding domain of Rhotekin, encoding the N-terminal 90 amino acids of Rhotekin (C21), or the Rac/Cdc42 binding CRIB domain (amino acids 56–272 of PAK1). HeLa cells were lysed in binding buffer (50 mM Tris pH 7.4, 150 mM NaCl, 5 mM MgCl_2_, 5 mM DTT, 1 mM PMSF, protease inhibitor complete (Roche), and 1% NP-40) at 4°C by sonification. The cell lysate was centrifuged at 13.200 ×g for 10 min, and the supernatant was incubated for 60 min with GST-C21(Rhotekin) (RhoA) and GST-PAK-CRIB (Rac1/Cdc42) immobilized to sepharose beads. Positive control was treated with 10 mM EDTA and 100 *μ*M GTP[*γ*S] at 30°C for 15 min to exchange the nucleotide. The complex of Rho protein and GTP[*γ*S] was stabilized by the addition of 60 mM MgCl_2_. Beads were washed with binding buffer. Bound Rho proteins were eluted by incubation in Laemmli lysis buffer at 95°C for 10 min and subjected to SDS-PAGE and Western blot analysis.

### 2.6. Western Blot Analysis

Proteins were separated by SDS-PAGE and transferred onto nitrocellulose membranes. Membranes were blocked with 5% non-fat-dried milk for 60 min. Subsequently, the membrane was incubated with primary antibody at 4°C over night, and secondary antibody conjugated with horseradish peroxidase for 1 h at room temperature. Blots were analysed by chemiluminescence reaction of ECL Femto using Kodak Image Station 440 CF.

### 2.7. Statistical Analysis

Statistical analysis was performed using Microsoft Excel and *P* values were analysed between two groups of data with a two-tailed student's *t*-test. *P ≤ 0.05; **P ≤ 0.01, ***P ≤ 0.001.

## 3. Results

### 3.1. CNF1-Induced Activation of Rho Proteins and Formation of FAs

**Figure 1:**
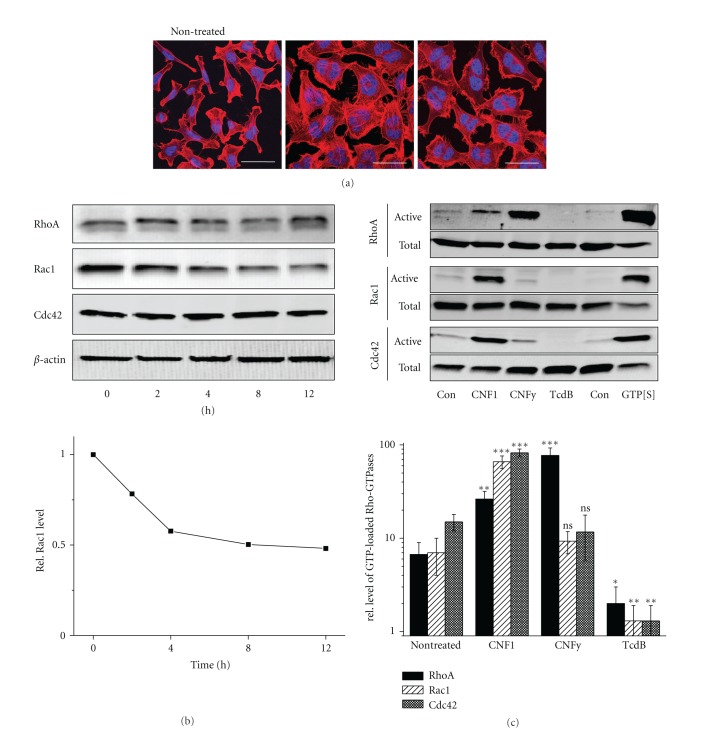
Formation of actin filaments upon activation of Rho proteins by CNF toxins. (a) HeLa cells were treated with CNF1 and CNFy for 24 h. The actin cytoskeleton and nuclei of CNF-treated HeLa cells was stained by rhodamine-phalloidin and DAPI, respectively. (b) HeLa cells were treated with CNF1 for 12 h. Reduced electrophoretic mobility of RhoA and the cellular levels of RhoA, Rac1, and Cdc42 were determined by Western blot analysis in time-dependent manner. Beta-actin was used as loading control. A representative Western out of three blots was presented. Signal intensities of Rac1 were quantified, and normalized to the level of beta-actin. (c) HeLa cells were treated with the indicated toxins for 5 h and the cellular levels of active GTP-bound RhoA, Rac1, and Cdc42 were determined by a pull-down assay using either GST-RBD (Rho-binding domain of Rhotekin) for RhoA and GST-PAK-CRIB (p21-binding domain of Pak1) for Rac1 and Cdc42. Total and precipitated Rho proteins were detected by immuno-blot analysis. A sample of lysates from nontreated cells was subjected to nucleotide exchange with the nonhydrolysable GTP*γ*S. Signal intensities (*N* = 3) were quantified, and normalized to the level of total Rho proteins. Results displayed are the mean ± SD of three independent experiments. *P* values <0.01 (**) and <0.001 (***) were considered as statistically significant as compared with nontreated cells.

The morphology of Hela cells was analysed upon treatment with either CNF1 or CNFy for 24 h. CNF1, that deamidates RhoA, Rac1, and Cdc4 induced pronounced formation of actin stress fibres, membrane ruffles and lamellipodia and filopodia, as visualized in cells stained with rhodamine-phalloidin (Figure 1(a)). CNFy, that deamidates RhoA, induced the formation of pronounced actin stress fibres, but the formation of membrane ruffles or filopodia was less pronounced, indicating that CNFy activated RhoA in HeLa cells (Figure 1(a)) [15]. RhoA deamidation results in inhibited contractile ring formation in cytokinesis; cells treated with either CNF1 or CNFy undergo cell cycling but omit cytokinesis [14]. Therefore, CNF1-/CNFy-treated cells were binucleated and exhibited an increased cell size (Figure 1(a)).

RhoA deamidation was tracked by a reduced electrophoretic mobility on SDS-PAGE [17]. RhoA exhibited reduced electrophoretic mobility upon 2 h of treatment with CNF1 (Figure 1(b)), indicating RhoA deamidation. Deamidated RhoA was present in CNF1-treated cells over a time period of 12 h (Figure 1(b)). CNFy comparably induced RhoA deamidation upon treatment for 2 h (data not shown). Deamidated Rac1 has been reported to be rapidly degraded in a proteasome-dependent manner [18, 19]. Rho protein expression was analysed in CNF1-treated HeLa cells by Western blot. The protein level of Rac1 was reduced, whereas the levels of RhoA or Cdc42 were almost constant in HeLa cells (Figure 1(b)), corroborating former observations [19].

To provide direct evidence on the activation of Rho proteins, the relative cellular concentrations of activated Rho proteins were determined using effector pull-down assay exploiting the Rho-binding domain of Rhotekin for RhoA and the PAK-CRIB domain for Rac1 and Cdc42. Upon CNF1 treatment, the cellular levels of RhoA-GTP, Rac1-GTP, and Cdc42-GTP were increased (Figure 1(c)). In contrast, the cellular level of selectively RhoA-GTP was increased in CNFy-treated Hela cells (Figure 1(c)). HeLa cells were further treated with Toxin B from *C. difficile* strain VPI10463 (TcdB), a broad-spectrum inhibitor of Rho proteins [20, 21]. In TcdB-treated cells, the levels of active RhoA-GTP, Rac1-GTP, or Cdc42-GTP were significantly reduced (Figure 1(c)), indicating that glucosylation of Rho proteins resulted in their inactivation. CNF1 thus activated RhoA, Rac, and Cdc42, while CNFy specifically activated RhoA.

### 3.2. Increased Cell-Matrix Binding upon Treatment with CNF1 and CNFy

**Figure 2:**
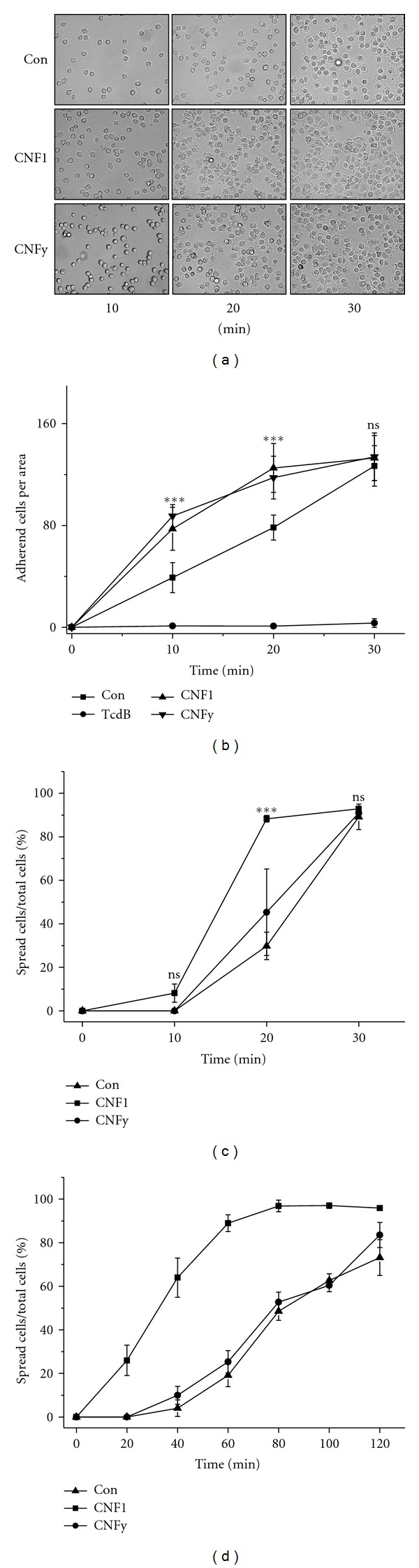
Increased cell attachment upon the activation of Rho proteins by CNF1 and CNFy. HeLa cells were treated with CNF1, CNFy, TcdB or buffer for 2 h and suspended by trypsin. Suspended cells were seeded onto a cell culture dish coated with fibronectin (a–c) or left uncoated (d). At the indicated time points, the medium containing nonadherent cells were removed. (a) Attached cells were documented by phase contrast microscopy. (b) The number of adherent cells per area was determined. Results displayed are the mean ± SD of three independent experiments (each experiment *n* = 400 cells). *P* values <0.001 (***) were considered as statistically significant as compared with nontreated cells. Cell spreading on fibronectin-coated polystyrol (c) or on noncoated polystyrol (d) was analysed in terms of the increasing number of spread per total cells. Results displayed are the mean ± SD of three independent experiments (each experiment *n* = 100 cells). *P* values <0.001 (***) were considered as statistically significant as compared with nontreated cells.

Following the hypothesis, that CNF1-induced activation of Rho proteins results in increased cell-matrix binding, the capability of suspended cells of establishing cell-matrix binding and cell spreading on fibronectin-coated polystyrol was analysed over time (Figure 2(a)). Suspended cells that bound to the matrix were initially rounded and then begin to spread out. Cells treated with either CNF1 or CNFy more efficaciously bound to the matrix compared to nontreated cells (Figures 2(a) and 2(b)). At later time points (≥30 min), cell-matrix binding of CNF-treated and untreated cells was comparable (Figure 2(b)). Cell-matrix binding was completely blocked upon inhibition of Rho proteins by TcdB (Figure 2(b)), confirming the critical role of Rho proteins in cell-matrix binding. Both CNF1 and CNFy thus increased the efficacy of cell-matrix binding. Next, the effects of CNF1 and CNFy on cell spreading were analysed. CNF1-treated cells more rapidly spread out compared to CNFy-treated or nontreated cells on fibronectin-coated polystyrol (Figure 2(c)), as quantified in terms of spread per total cells. Cell spreading was further analysed on a polystyrol matrix. Cell spreading on polystyrol was clearly delayed compared to spreading on fibronectin-coated polystyrol (Figures 2(c) and 2(d)). CNF1 increased cell spreading also on the polystyrol matrix, while cell spreading of CNFy-treated cells was comparable to nontreated cells (Figure 2(d)). Cell spreading was specifically triggered by CNF1 (not CNFy), which correlated with the capability of CNF1 of activating Rac1 (Figure 1(c)).

**Figure 3:**
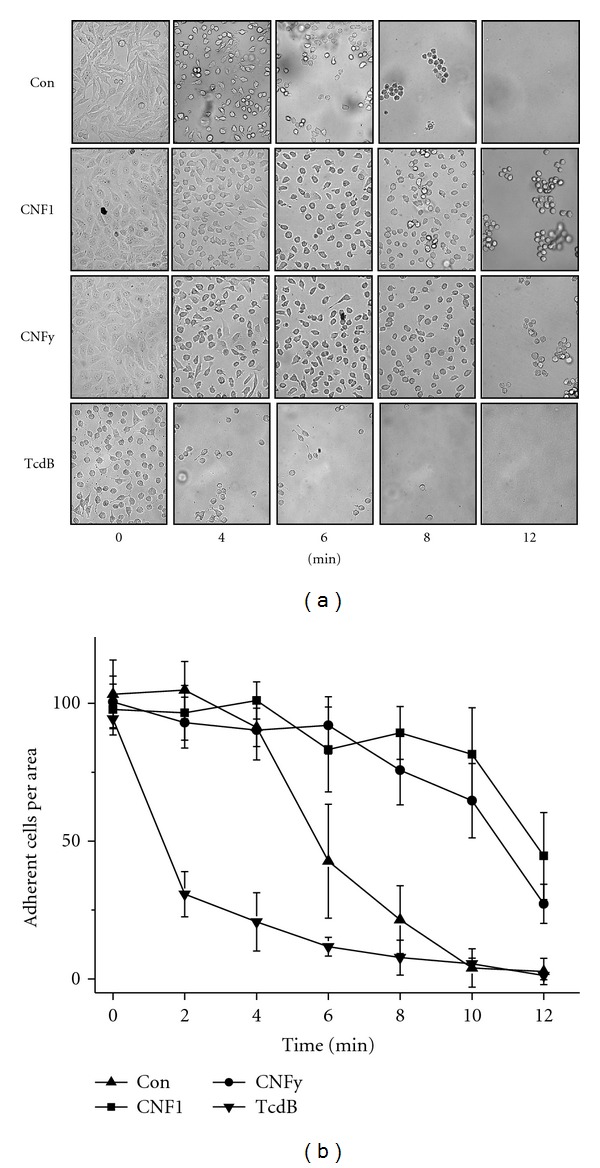
Reduced susceptibility of CNF1/CNFy-treated cells to trypsin-induced cell detachment. Adherent HeLa cells were treated with CNF1, CNFy, or TcdB for 2 h. Cells were incubated with trypsin for the indicated time and the medium with suspended cells was removed. (a) Adherent cells were documented by phase-contrast microscopy. (b) The number of adherent cells per area was determined in a time-dependent experiment. Results displayed are the mean ± SD of three independent experiments (each experiment *n* = 100 cells).

The consequences of the CNF-induced activation of Rho proteins to cell-matrix adhesion were further analysed in terms of the susceptibility of cells to trypsin-induced cell detachment. Upon treatment with CNF1 or CNFy (less pronounced), trypsin-induced cell detachment was delayed (Figures 3(a) and 3(b)). In contrast, inhibition of Rho proteins by TcdB strongly increased the susceptibility towards trypsin-induced cell detachment, showing that Rho proteins were required for the establishment of cell-matrix adhesions (Figures 2(a)–2(d)). Treatment with CNF1 or CNFy triggered cell-matrix binding and increased the persistence of pre-established cell-matrix adhesion.

### 3.3. Formation of Focal Adhesions Induced by CNF1

**Figure 4:**
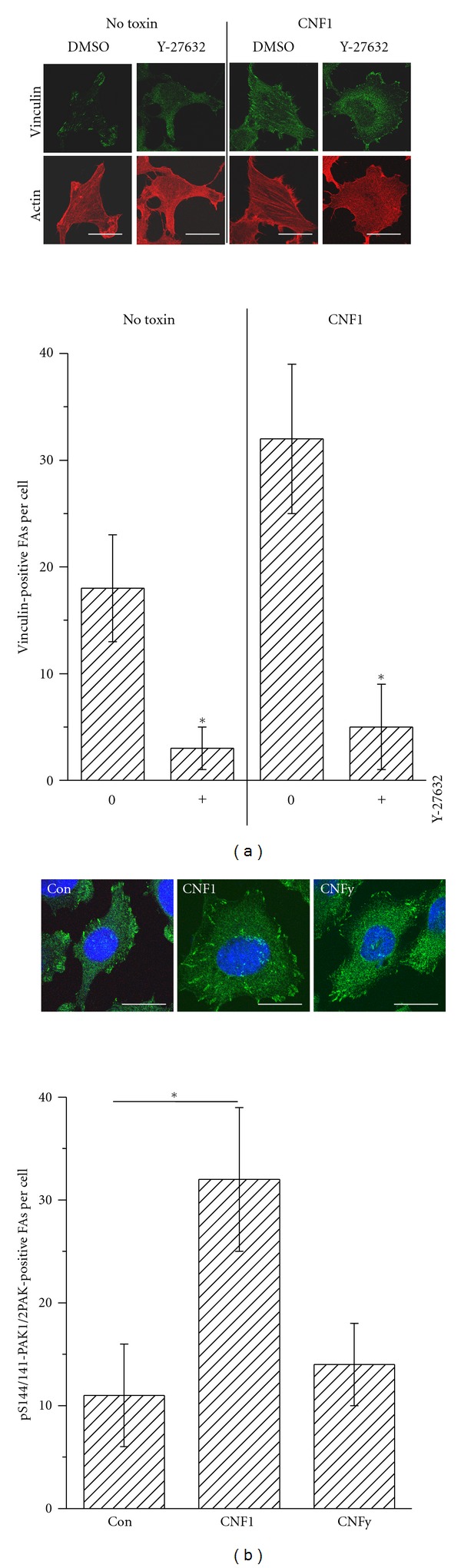
CNF1/CNFy-induced formation of focal adhesions. Focal adhesions in HeLa cells treated with the indicated CNF toxins or buffer were stained for the FA marker vinculin (a) or pS144/141-PAK1/2 (b) using immunocytochemistry. The number of vinculin-positive (a) or pS144/141-PAK1/2-positive FAs (b) per total cells was analysed in three independent experiments (each experiment *n* = 30 cells). *P* values <0.05 (*) were considered as statistically significant as compared with nontreated cells.

The transition of cell-matrix adhesions from the initial punctate focal complexes into the mature elongated form, the focal adhesions (FAs), has been attributed to the activity of Rho proteins [22, 23]. To check if CNF1 induced FA formation, HeLa cells were stained for actin and vinculin, with the latter being an established FA marker. Elongated structures with a longitudinal length of ≥1.5 *μ*m were considered as FAs. The number of FAs per cells strongly increased upon treatment with CNF1 (Figures 4(a) and 4(b)). Thereby, CNF1-induced formation of stress fibres and FAs was completely abolished upon treatment with the ROCK inhibitor Y-27632. RhoA-ROCK signalling was critical for FA formation in nontreated as well as in CNF1-treated cells. Furthermore, ROCK inhibition by Y27632 induced cell spreading in CNF1-treated cells (Figure 4(a)).

Pronounced cell spreading upon initial cell-matrix binding was exclusively observed in CNF1- (not in CNFy-) treated cells, suggesting that cell spreading depended on Rac1/Cdc42 activation rather than on RhoA activation. Next, the activity of the Rac1/Cdc42 effector protein PAK, another FA component [5], was analysed in FAs exploiting a phosphospecific antibody recognizing pS144/141-PAK1/2, the activated form of PAK1/2 (Figure 4(b)). CNF1 induced a pronounced increase of active PAK-positive FAs compared to nontreated or CNFy-treated cells. (Figure 4(b)). This observation showed that CNF1 (not CNFy) activated PAK1/2, reflecting specific activation of Rac1/Cdc42 by CNF1. Cell spreading thus correlated with the pronounced increase of PAK-positive FAs in CNF1-treated cells.

### 3.4. Reduction of Directional Cell Migration upon Activation of Rho Proteins by CNF1 and CNFy

**Figure 5:**
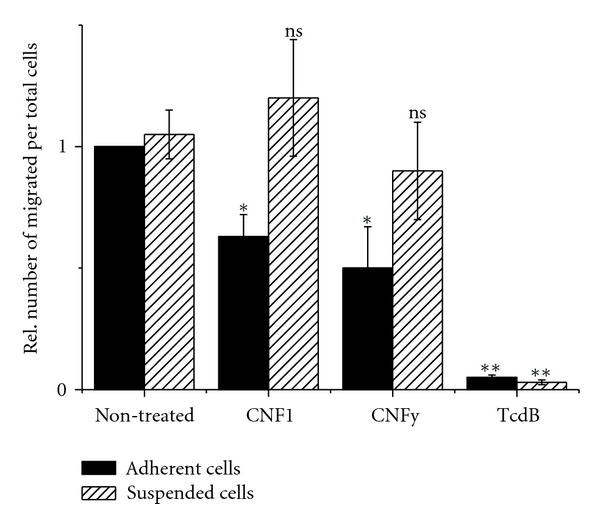
Reduced cell migration of adherent cells upon treatment with CNF1 and CNFy. Migration of HeLa cells was analysed in a Boyden chamber experiment. Cell migration was induced by a serum gradient of 10% bovine calf serum for 5 h. Cell migration was quantified as the number of cells that passed bottom membrane. Suspended cells: HeLa cells were pretreated with the indicated toxins for 2 h. Cells were suspended by trypsin, directly seeded onto the membrane and the serum gradient was applied for 5 h. Adherent cells: HeLa cells were seeded on the membrane and allowed to attach. Cells were then treated with the indicated toxins for 2 h. Afterwards the serum gradient was applied for 5 h. Cells on the upper membrane surface were scraped with a cotton swab, and cells on the bottom surface stained with Hoechst 33342. After incubation at 37°C for 15 min, cells were analyzed by fluorescence microscopy using a Zeiss Axiovert 200 M (excitation 365 nm, emission 420 nm). Five adjacent microscope fields for each membrane were counted at ×20 magnification. Results displayed are the mean ± SD of three independent experiments. *P* values <0.05 (*) and <0.01 (**) were considered as statistically significant as compared with nontreated cells.

Finally, the hypothesis was followed that increased cell-matrix adhesion affects the migration of CNF-treated cells. HeLa cells were treated with the CNF toxins for 2 h and then allowed to adhere on a transwell membrane. Cell migration was stimulated using a serum gradient (Boyden chamber transwell migration experiment). Under these conditions, about 50% of nontreated cells migrated through the membrane within a time period of 5 h (data not shown). TcdB treatment completely blocked migration of adherent cells (Figure 5). Cells either treated with CNF1 or CNFy migrated to some extent. The efficacy of migration was reduced compared to nontreated cells (Figure 5). Furthermore, the migration of suspended cells was analysed. Therefore, adherent cells were pretreated with the toxins for 2 h, suspended by trypsination, placed onto the Boyden chamber membrane, and directly allowed to migrate. Under this condition, about 50% of nontreated cells migrated through the membrane within a time period of 5 h (data not shown). Cell migration of adherent and suspended cell through the membrane was thus comparable. The migration of suspended cells was completely blocked upon TcdB treatment (Figure 5). Interestingly, treatment of suspended cells with either CNF1 or CNFy did not affect cell migration (Figure 5). Suspended cells pretreated with CNF1 or CNFy migrated with an efficacy comparable to that of nontreated cells (Figure 5). Deamidation of Rho proteins thus reduced cell migration under the condition of pre-established cell-matrix adhesion.

## 4. Discussion

In this study, the effects of Rho-modifying toxins on cell adhesion are analysed to dissect distinct roles of Rho proteins in cell adhesion. In particular, the application of the RhoA-specific CNFy is exploited to dissect the role of RhoA activation to the observed effects. The initial step of cell adhesion is cell-matrix binding. Cell-matrix binding is strongly increased by both CNF1 and CNFy to comparable extent, showing that increased cell-matrix binding of CNF1-/CNFy-treated depends on RhoA deamidation. Cell matrix-binding of TcdB-treated cells is completely prevented, showing that the activity of Rho proteins is required for cell-matrix binding. Cell-matrix binding is followed by cell spreading. Cell spreading is specifically triggered by CNF1 (not CNFy), suggesting that cell spreading rather depended on Rac1/Cdc42-dependent rather than RhoA-dependent signaling pathways. Furthermore, the RhoA effector protein ROCK seems to negatively regulate cell spreading, as cell spreading of CNF1-treated cells is increased upon inhibition of the ROCK by Y-27632.

Cell-matrix binding and cell spreading include the formation of focal contacts, which maturate into elongated focal adhesions (FAs) [22, 23]. Activation of Rho proteins by CNF1 strongly increased the number of vinculin-positive FAs per cells. The FAs formation depends on RhoA/ROCK signalling in CNF1- and nontreated Hela cells, as ROCK inhibition by Y-27632 completely blocks FAs formation. The latter observation is consistent with the view that the elongated form of FAs depends on the presence of stress fibres, which formation is inhibited upon Y-27632 treatment [4]. The increased number of FAs in CNF1-treated cells (compared to nontreated cells) likely results from CNF1-mediated activation of Rho proteins, which reportedly drives the maturation of focal contacts into focal adhesions [22, 23]. The Rac1/Cdc42 effector protein PAK1/2 is also regarded as a component of FAs [24]. In CNF1- (not CNFy-) treated the number of pS144/141-PAK1/2-positive FAs, that is, FAs with activated PAK1/2, is strongly increased. This observation reflects the fact that specifically CNF1 (not CNFy) activates Rac1/Cdc42. Interestingly, active PAK has been suggested to promote FA disassembly and cell detachment [3, 24]. Although active PAK is present at the FAs of CNF1-treated cells, these cells exhibited a reduced susceptibility of cells to trypsin-induced cell detachment compared to CNFy-treated and nontreated cells. The activation of RhoA-dependent and other Rac1/Cdc42-dependent pathways that positive regulates cell attachment (including those regulating cell spreading) obviously overbalances cell detachment driven by active PAK1/2 in CNF1-treated cells. CNFy-treated cells exhibit a reduced susceptibility of cells to trypsin-induced cell detachment compared to nontreated cells, showing that the activation of RhoA-dependent pathways is sufficient for increased cell adhesion.

The consequences of increased cell adhesion of CNF1-/CNFy-treated cells on cell migration is differentially analysed in adherent and suspended cells. Suspended cells treated with either CNF1 or CNFy migrated to an extent comparable to that of nontreated cells. Thus the presence of deamidated Rho proteins *per se* did not inhibit cell migration. In contrary, microinjection of Rac1-Q61L is a well-established method to trigger cell migration [25]. This may explain, why migration of CNF1-treated suspended cells is slightly (not significantly) increased. In contrast, the migration of suspended cells was completely blocked upon TcdB pre-treatment. This observation illustrates that activity of Rho proteins and cell-matrix adhesion are both prerequisites for cell migration [4]. The matrix adhesion of suspended TcdB-treated cells is completely blocked, based on the fact that the activity of Rho proteins is required for cell-matrix adhesion. The analysis of cell migration of adherent cells revealed that migration of CNF1-/CNFy-treated cells is reduced. Against the background that the deamidation of Rho protein *per se* does not reduce cell migration, this reduction most likely reflects that increased cell adhesion impairs cell migration. Cell migration includes FA disassembly and loss of cell adhesion at the cell rear [6]. This process mainly depends on RhoA and correlates with the observation of this study that both CNF1 and CNFy reduce cell migration. RhoA deamidation may thereby prevent FA disassembly and loss of cell adhesion at the cell rear of adherent cells, which remains to be analysed in detail. In conclusion, CNF1 and CNFy both reduce cell migration specifically under the condition of pre-established cell-matrix adhesion. This observations makes CNF toxins an interesting tool, as it allows the dissection of the role of Rho proteins in cell adhesion (CNF sensitive) from the role of Rho proteins in other processes (CNF insensitive) of the migrating cells.

In adherent epithelial cells, integrin-mediated cell survival is promoted through several pathways including the phosphatidylinositol 3-kinase (PI3K)-Akt pathway [26]. Epithelial detachment of epithelial cells results in decreased survival signalling. The detached cells undergo a type of apoptotic cell death, also referred to as anoikis [27]. Against this background, the capability of CNF1 of inducing increased cell adhesion is of particular interest. CNF1 has been presented to preserve epithelial cells from apoptosis induced by various stimuli [13, 28, 29]. Two distinct mechanisms thereby may be responsible for the antiapoptotic activity of CNF1: (i) activation of Rac1/Cdc42 suppresses apoptosis in a PI3K/Akt-dependent manner [27, 29, 30]; (ii) increased cell adhesion preserves cells from detachment and subsequent anoikis [13, 28]. Given that increased cell adhesion is sufficient for preserving the detachment of epithelial cells, CNFy should exert antiapoptotic activity as well, an aspect of the activity of CNFy that remains to be investigated.

## Abbreviations

CNF: Cytotoxic necrotizing factor,
FA: Focal adhesion,
PAK: p21-activated kinase,
TcdB: Toxin B from *C. difficile* strain VPI10463.
